# Anonymous view on transgender soldiers: content analysis of online news headlines and comments in South Korea

**DOI:** 10.1186/s12889-022-14565-z

**Published:** 2022-11-15

**Authors:** Jeehye Lee, Dong-Hee Ryu, Su-Jin Lee

**Affiliations:** 1 Department of Preventive Medicine, Eulji University School of Medicine, Daejeon, 34824 Republic of Korea; 2 Present address: National Emergency Medical Center, National Medical Center, Seoul, 04564 Republic of Korea; 3 Department of Preventive Medicine, Daegu Catholic University School of Medicine, 33 Duryugongwon-ro 17-gil, 42472 Daegu, Republic of Korea; 4 Public Policy Team, Daegu Public Health Policy Institute, 41940 Daegu, Republic of Korea

**Keywords:** Military personnel, Newspaper article, Sexual and gender minorities, Transgender persons

## Abstract

**Background:**

The inclusion of transgender soldiers in the military service raises a fundamental question about the dichotomous categorization of human sexes based on anatomy and gender role within a specialized organization where the most masculine is commonly accepted. In March 2021, Hee-Soo Byun, the first transgender soldier in Korea to come out in public, and who was forcefully discharged after gender affirming surgery, died by suicide. With no anti-discrimination laws, the cultural background of the Korean society hardly creates an LGBT (Lesbian, Gay, Bisexual, and Transgender) — friendly environment and shows a negative attitude towards gender minorities.

**Methods:**

A total of 193 online news article headlines were analyzed, and 1046 comments were categorized inductively based on the presented rationales.

Results: Before Byun’s public appearance, the frequent use of provocative expressions, which could evoke prejudice and discrimination, was found in published article headlines. Of the 724 comments that presented opinions on transgender soldiers, approximately 75% opposed Byun serving in the military in any form, including as a female soldier.

**Conclusions:**

This study aimed to investigate online news articles and the comments regarding Byun’s case to estimate the acceptability of transgender people serving in the military. The results of this study are expected to serve as a basis for the formulation of policies that protect the human rights of transgender people.

## Background

On 22 January 2020, an active-duty soldier, staff sergeant Hee-Soo Byun came out in public as a trans woman followed by a historical decision of the Korean military [1]. By holding on to her childhood dream of becoming a soldier, Byun endured the difficulties caused by gender dysphoria and underwent gender affirming surgery [1]. The military imposed a compulsory discharge due to “physical and mental disability” because Byun underwent sex reassignment surgery on an official vacation [1]. The South Korean military policy strictly bans transgender people from serving in the military and deemed the gender affirming surgery as an ‘intentional loss of testicles and penis.’ The military imposed a compulsory discharge even though Byun wished to continue to serve as a female soldier. Furthermore, Byun’s petition for reinstatement was rejected as well. On 11 August 2020, Byun declared filing a lawsuit for the cancellation of the discharge, stating that “I expect the judiciary to make a righteous decision and the Republic of Korea to overcome hatred [1].” Unfortunately, Byun was found dead on 3 March 2021, taking her own life [2].

Byun is documented as the first openly transgender soldier who was forcefully discharged, and this case sparked a social debate about transgender people’s right to self-determine their occupations. Article 39 of the Constitution of the Republic of Korea declares that “*All citizens shall have the duty of national defense* [3],” and Article 3 of the Military Service Act states that “*Every man of the Republic of Korea shall faithfully perform mandatory military service. A woman may perform only active service or serve through volunteering* [4].” Although trans women can be exempt from military service with permission for legal gender affirmation these days, proof of a completion of genital surgery used to be required along with the legal process to qualify for an exemption previously [5]. However, there is no relevant policy or appropriate medical services for trans women who are willing to serve the country. This means that their right to serve is not guaranteed, even though the Constitution guarantees the right to pursue happiness, freedom of occupation, and prohibits discrimination [3]. The United Nations High Commissioner for Human Rights condemned Byun’s forceful discharge and stated that “the dismissal of Ms. Byun would violate the right to work and the prohibition of discrimination based on gender identity under international human rights law [2].”

Korea is a conservative country and less tolerant toward the LGBT (Lesbian, Gay, Bisexual, and Transgender) community as compared to other countries. Such characteristic is commonly considered as rooted in the Confucian ideology that emphasizes concepts such as traditional conservatism and relational hierarchy [6, 7]. There is no anti-discrimination law in Korea even with previous eight proposals to enact the law since 2007. It was previously reported that negative beliefs, attitudes, and fear toward transgender people developed into a social stigma that gave rise to prejudices, discrimination, and even violence against transgender people [7]. Yi and Phillips pointed out that until recently, the LGBT community was not usually included in discussions on human rights issues in Korea [8]. The same can also be seen in academic fields. A 2017 study found that among 32 studies on transgender health conducted in Korea, only five dealt with the social health of transgender people, while most focused on the clinical examination and surgical experiences of transgender individuals [5]. It is essential to discuss the bias and discrimination that transgender people experience in their occupational fields as it may reflect the level of impact on their social health. This is especially true for mental health status and unequal employment opportunities among transgender people [9]. It was previously reported that transgender people are more vulnerable to suicide risks and mental health problems such as substance abuse or posttraumatic stress disorder [7]. Such mental health problems occur not because someone is transgender but because of the bias, stigma, and discrimination transgender people experience from society. Considering the case of Byun, research on occupational discrimination and job opportunities for transgender soldiers in Korea is necessary.

In March 2021, the Defense Minister publicly mentioned the necessity of researching the inclusion of transgender people in military service [10], but no such study concerning Byun’s case has been published yet. This study focused on the social perspective on transgender soldiers rather than the transgender individuals because the social impact of the Byun’s case highlighted the necessity to discuss the acceptability of transgender soldiers in Korea for the first time. The objective of this study was to determine the acceptability of transgender people serving in the military by investigating online news articles and readers’ comments regarding Byun’s case. In addition, as part of the analysis, we examined online news article headline keywords whether the media would discuss Byun’s experience of gender dysphoria.

## Methods

### Sample

**Fig. 1:**
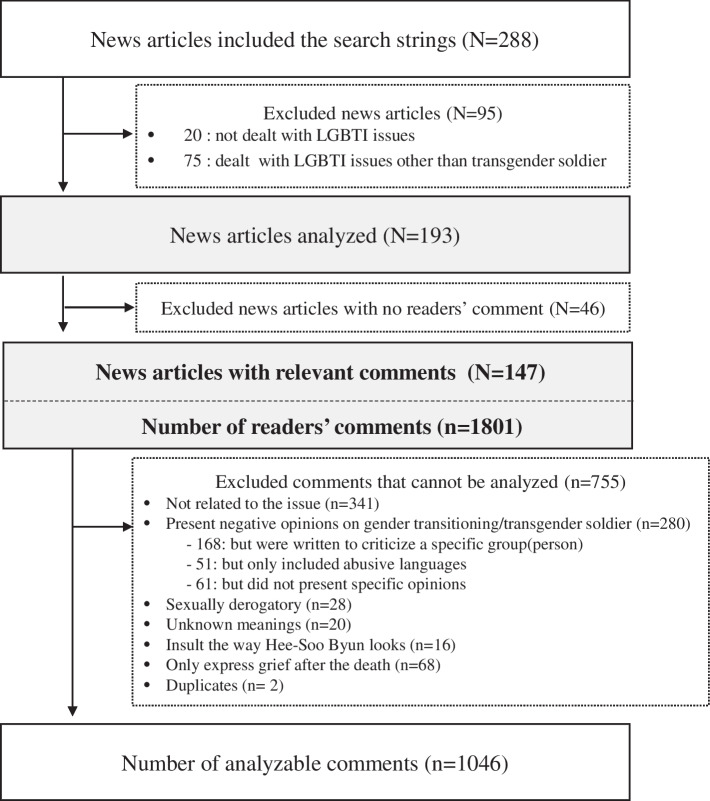
Sample selection process

The study included four major daily newspapers that were published online in South Korea from 1 January 2020 to 31 May 2021. The political orientation and number of readers of newspapers was considered in the sample selection, and two conservative (Chosun Ilbo and Joongang Ilbo) and two liberal (Hankyoreh and Kyunghyang Shinmun) newspapers were selected. News articles were selected using the following search strings: Hee-Soo Byun, (staff) sergeant Hee-Soo Byun, former (staff) sergeant Hee-Soo Byun, (staff) sergeant Byun, former (staff) sergeant Byun, transgender soldier, forceful discharge. A total of 288 articles were selected and those which simply mentioned Hee-Soo Byun but not the issues of transgender soldiers were eliminated from the analysis. Such excluded articles introduced a LGBT film, an admission of a trans woman to a women’s university, and other political issues. A total of 193 online news article headlines were analyzed, and 147 articles were analyzed for readers’ comments (Fig. 1).

### Analysis of headline keywords

Headline keywords depicting Byun were extracted manually using the Korean morpheme dictionary (NIADic) distributed by the National Information Society Agency. Numbers, dates, and postpositions (suffixes or short words that immediately follow a noun or pronoun) included in the headlines were not collected. Since news articles were originally written in Korean, each author translated the extracted keywords into English one by one and confirmed them altogether. To guarantee the quality of English translation, the translated words were retranslated by a bilingual English native speaker into Korean and checked. The date and time of publication were checked, and news articles were further categorized into ‘anonymous (before 22 January 2020)’, ‘came out in public (from 22 January 2020 to 3 March 2021)’, and ‘dead (since 3 March 2021)’ based on Byun’s timeline. For the ‘anonymous’ and ‘came out in public’ groups, keywords used to depict Byun were examined thoroughly.

### Analysis of readers’ comments

It was ensured that all four newspapers only allowed registered website members, aged 14 years and above, to leave comments. The commenters’ private information, including their names, was not provided. Each comment was treated as an individual response even if they were presumed to be posted by the same commenter. Any comments containing typos, symbols, slangs, or buzzwords with uncertain meanings were not entirely excluded: instead, the content of the comment was considered and used for analysis. All comments presenting opinions on transgender soldiers or surgical transition were identified, and comments not suitable for analysis (comments that criticize a specific group (person), that only contain abusive or sexually derogatory languages, or that present simple mourning reactions, etc.) were excluded. A total of 1046 comments were analyzed. Each author explored the contents of the comments, classified the comments into those that are in favor of or against transgender’s inclusion in the military service, and further categorized comments inductively based on the presented rationale. All authors reviewed and confirmed the categorization.

### Software

Microsoft Excel (Microsoft Corporation, Redmond, WA, USA) was used for all analyses and graphic production.

### Ethical statement

The ethical evaluation was completed by the institutional review board of Daegu Catholic University Medical Center (CR-21-123-PRO-001-R) and further evaluation was exempted since publicly available data was used.

## Results

**Fig. 2:**
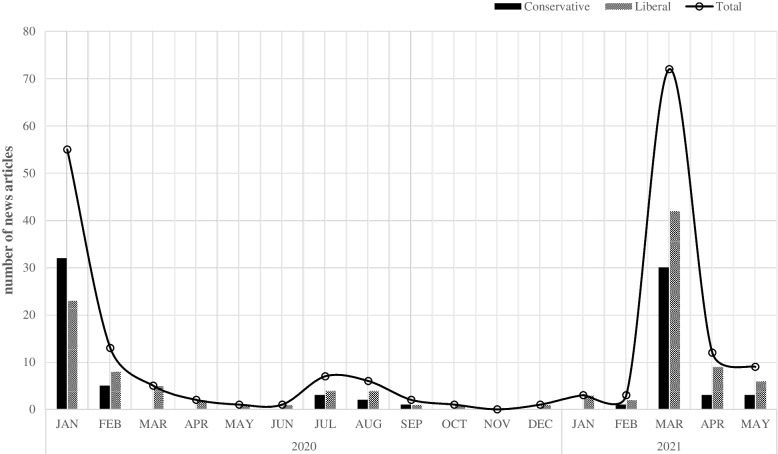
Number of news articles published according to political orientation of newspapers

Figure 2 represents the number of news articles, according to the political orientation of newspapers, dealing with Byun’s case that were published from January 2020 to May 2021. The number of news articles published in liberal newspapers per month was higher than that of conservative newspapers except for January 2020. In March 2021, when Byun was found dead, the total number of articles published was 72 (conservative: 30; liberal: 42).

Table 1 shows headline keywords used to describe Byun before coming out in public. Most of the articles reported during this period described Byun in their headlines with specific military rank and position. The terms “on vacation” and “on duty” were used in seven headlines. Table 2 shows headline keywords used to describe Byun after the forceful discharge. Of 65 articles, 12 used the term “former.”

**Table 1:** Headline keywords used to describe Byun before coming out in public (before 22 January 2020)

Newspapers	Total number of articles	Descriptions
A	8	gender transitioned soldier (3), gender transitioned non-commissioned officer (1), gender transitioned staff sergeant (1), gender transitioned tank driver (1) male non-commissioned officer who received sex reassignment surgery on vacation (1), male staff sergeant who is a tank driver (1)
B	16	transgender non-commissioned officer (6), the first transgender staff sergeant in Korean military (1), transgender staff sergeant (1), transgender (1) gender transitioned non-commissioned officer (2), staff sergeant transitioned gender on duty (1), staff sergeant transitioned gender on vacation (1) non-commissioned officer transitioned gender on vacation (2) gender transitioned male non-commissioned officer (1)
C	4	transgender soldier (1), first transgender soldier (1)
		male non-commissioned officer transitioned gender on vacation (1), male non-commissioned officer (1)
D	7	gender transitioned soldier (3), military sexual minority (1), non-commissioned officer with sex reassignment surgery (1)
		army non-commissioned officer (1), “A” sergeant (1)

Numbers in the parentheses indicate the number of articles fall into the category

**Table 2:** Army rank descriptions after the forceful discharge (from 22 January 2020 to 2 March 2021)

Newspapers	Total number of articles	Descriptions
A	9	former staff sergeant (4)
		staff sergeant (4)
		soldier (1)
B	11	former staff sergeant (2)
		non-commissioned officer (4), staff sergeant (2), soldier (1)
		not specified (2)
C	26	former staff sergeant (4)
		staff sergeant (9), soldier (3), non-commissioned officer (1)
		not specified (9)
D	19	former staff sergeant (2)
		staff sergeant (9), non-commissioned officer (2), soldier (1)
		not specified (4), Mr. (1)

Numbers in the parentheses indicate the number of articles fall into the category

Table 3 shows the types of newspaper readers’ comments on transgender soldiers according to their opinions on surgical transition. Of 1046 comments, 549 comments opposed transgender soldiers, and 15.5% of them demonstrated negative opinions on surgical transition. However, 175 comments were supportive of transgender soldiers, and the proportion of those who believed that surgical transition is one’s free will was 26.9%. Yet, there was only one comment with supportive opinions on both transgender soldiers and surgical transition. Of 308 comments with no opinions on transgender soldiers, 63.3% demonstrated negative opinions on surgical transition.

**Table 3:** Readers’ comments on transgender soldiers according to opinions on surgical transition

Category		Opinions on transgender soldiers				
		Supportive	Opposing	Undecided^a^	Undefined	Subtotal
**Opinions on surgical transition**	**Supportive**	1 (0.57)	1 (0.18)	0 (0.00)	2 (0.65)	4 (0.38)
	**Opposing**	5 (2.86)	85 (15.48)	0 (0.00)	195 (63.31)	285 (27.25)
	**Not opposing (one’s free will)**	47 (26.86)	34 (6.19)	0 (0.00)	111 (36.04)	192 (18.36)
	**Undefined**	122 (69.71)	429 (78.1)	14 (100.0)	–	565 (54.02)
	**Subtotal**	175 (16.73)	549 (52.49)	14 (1.34)	308 (29.44)	1046 (100.0)

^a^Undecided: presented comments that opinions of cisgender female soldiers should be taken into consideration

Some examples of the newspaper readers’ supportive and opposing comments on transgender people are presented in Table 4 and Table 5. The proportion of supportive comments that presented opinions based on no specific personal rationale was 4.6%, while that of opposing comments was 18.4%. However, most of the analyzed comments presented opinions based on personal rationale. The first issue was Byun’s eligibility as a soldier. About 8% of comments opposing the inclusion of transgender people in military service stated that Byun’s physical characteristics (completion of genital surgery and XY sex chromosome) or mental characteristics (gender dysphoria) indicate inappropriateness in continuing service based on military regulations. While some comments in favor of transgender soldiers did not agree with the implication that gender affirming surgery would negatively influence Byun’s ability as a soldier, about 25% of supportive comments addressed the necessity of proving Byun’s current suitability as a female soldier following the gender affirming surgery. The second issue was compliance with employment contracts and the legitimate employment process. Of all the opposing comments, 27.1% emphasized that the gender affirming surgery caused a termination of an employment contract or should be interpreted as noncompliance in the workplace. Some comments that presumed the possibility of Byun serving as a female soldier also mentioned that reexamination is required due to the change in the premise of Byun being a man at the time of joining the military. The third issue was human rights. While 10.3% of all supportive comments backed Bun’s human rights as a trans woman, 24.4% of all opposing comments emphasized the human rights of her female and male colleagues. Some opposing comments presented worrisome opinions expressing the risk of violating the human rights of cisgender female soldiers in taking a shower, getting dressed, and sleeping if they were to stay with a trans woman, while supportive comments addressed no problems. Additionally, some comments presented concerns that the human rights of cisgender male soldiers would be in danger; considering that Byun was a tank driver, which is a position appointed to male soldiers in South Korea, allowing a trans woman to be trained with male soldiers would limit the men’s freedom in dressing, urination/defecation, and encampment.

**Table 4:** Newspaper readers’ supportive comments on the inclusion of transgender people in the military service

Themes (N)	Opinions/Rationales	n (%)	Representative quotes
I. Eligibility (*N* = 53)	1. It is possible if she passes the screening process as a woman and start at square one because her current eligibility as a female soldier should be tested after the gender reassignment surgery.	43 (24.6)	*· It is pretty logical to confirm Byun’s eligibility as a female soldier.* *· Male and female non-commissioned officers undergo different screening processes. Therefore, Byun has to apply as a female soldier.*
	2. She should not be dismissed if eligible as a soldier (should consider her ability, not gender)	10 (5.7)	*· If there’s no setback as a tanker, Byun should not be dismissed.* *· As long as Byun had outstanding abilities as a soldier, there would be no problem.*
II. Compliance with employment contract (*N* = 50)	1. It is possible if she passes the screening process as a woman and start at square one because she was originally tested as a male so the gender reassignment was a breach of contract.	49 (28.0)	*· The military is not a place for fun. Since Byun’s eligibility as a soldier was tested as a male, Byun must undergo re-examination after the gender reassignment surgery.* *· Byun was enlisted as a man. It is a breach of contract to ask to serve in the military as a woman even after having had gender reassignment surgery. Byun is making demands that is difficult for the military to accept.*
	2. It is possible if she passes the screening process as a woman and starts at square one following a proper punishment because gender reassignment surgery is damaging ordnance (part of a soldier’s body)	1 (0.6)	*· If not ashamed, Byun should have tried as a female soldier after receiving punishment for the crime of damaging military supply.*
III. Human rights (*N* = 18)	1. Byun’s free will should be respected	8 (4.6)	*· There’s no reason to kick out Byun when willing to serve.* *· Article 11 of the Constitution of the Republic of Korea ① All citizens are equal before the law. No one shall be discriminated against in all areas …*
	2. Any discrimination against transgender people happens in less developed countries	6 (3.4)	*· An advanced society that recognizes and respects diversity is still a long way off!!* *· What kind of a society can discuss industrial revolution and enhance birth rate when transgender soldiers are not allowed?*
	3. There’s no (scientific) evidence to ban transgender soldier	4 (2.3))	*· … Does gender reassignment do any harm to the military? It would not be too late to dismiss after proving the harmfulness.* *· … There’s no studies or data about how services of transgender people affect military combat ability …*
IV. Illegitimate employment process (*N* = 17)	1. It is possible if she passes the screening process as a female and start at square one because that would be unfair to her female colleagues because screening process for female noncommissioned officers is more competitive than male’s.	13 (7.4)	*· Screening process for female non-commissioned officers is more competitive than male. I wonder if Byun was worried about failing the test when applying as a female soldier. Otherwise, Byun could have tried for the exam as a female after the gender reassignment surgery.* *· I don’t think the forceful discharge is wrong. The rate of competition among females is higher …*
	2. It is possible if she passes the screening process as a female and starts at square one following a legal process to become a female^a^	4 (2.3)	*· I am in favor of Byun’s enlisting as a female soldier after she becomes a woman by a law. Even from a technical point of view, it would be impossible to register her as a female soldier without undergoing a proper legal process.*
V. Conceptional matter (*N* = 16)	1. She can be treated as a female soldier	11 (6.3)	*· I think Byun can be a great female soldier. It may be a little uncomfortable at first, but people can get used to it over time.* *· Since there is the same position in a female troop, the military should let Byun serve as a female.*
	2. It’s a matter of revising law, regulation, or social perspectives	5 (2.9)	*· The military must have taken discharge measures based on the legal basis, but this happened. It’s because the military regulations are wrong, or they have not kept up with changing times.* *· We need to change the system so that gender identity does not become a discriminatory factor in maintaining a job.*
VI. National perspectives (*N* = 9)	1. Byun’s patriotism should be respected.	6 (3.4)	*· Even if you don’t like her, respect her as a soldier who wants to defend this country …* *· Byun is willing to sacrifice for the country. Then why not?*
	2. The forceful discharge is a national loss	3 (1.7)	*· Don’t waste time and money used for training.* *· I don’t understand this discharge because there are not enough soldiers due to the population decrease.*
VII. Undefined (*N* = 12)	1. Presented no specific rationale	8 (4.6)	*· I look forward to Byun’s return to the military.* *· Jut let Byun work in the original position.*
	2. Presented multiple rationale	4 (2.3)	*· Byun wants to serve in the military, then why not? It takes a lot to train a tank gunner.* *· When changed from a male to a female, then you simply become a female soldier. … Everyone is born with the fundamental right to pursue happiness. If the person’s choice is not harmful to others, the society has no right to force or restrain one’s own will.*
**Total**		**175 (100.0)**	

^a^Byun had completed the legal process to formally become a woman on 10 February 2020

**Table 5:** Newspaper readers’ opposing comments on the inclusion of transgender people in the military service

Themes (N)	Opinions/Rationale	n (%)	Representative quotes
I. Compliance with employment contract (*N* = 149)	1. Undergoing gender reassignment surgery on duty is a violation of obligations of soldiers	35 (6.4)	*· It’s not a human rights issue. It’s a matter of military discipline, morale, and regulations.* *· Isn’t it a loss of military power?*
	2. Undergoing gender reassignment surgery on duty is a breach of contract	32 (5.8)	*· You really don’t know what the problem is? The military hired a person with man’s power and physical condition, and it surely is a breach of contract.* *· The Ministry of National Defense had recruited a male soldier. Recruitment guidelines are part of the contractual relationship.*
	3. She should be punished for military disobedience	24 (4.4)	*· I am opposed to the dismissal. Byun should be arrested for damaging military supply and disobedience.* *· A soldier with this kind of spirit who behaves like this should be treated according to military law.*
	4. Undergoing gender reassignment surgery on duty is a military disobedience (there was no permission by supervisors)^a^	23 (4.2)	*· Isn’t it your fault that you had sex reassignment surgery on vacation without any permission when serving in the military? It makes no sense to embrace and accept one who changed gender on duty.* *· What do you want after breaking the military law? Soldiers need permission from the commander to go abroad.*
	5. Undergoing gender reassignment surgery on duty was inappropriate and she should’ve done it before joining the Army or after self-dismissal	21 (3.8)	*· Byun should’ve had gender reassignment surgery after being discharged from the military or before joining. Did Byun want to get attention from the media?* *· You don’t stay in the military for the rest of your life. Shouldn’t Byun change after self-dismissal? So sad …*
	6. She should be punished for damaging ordnance (part of a soldier’s body)	11 (2.0)	*· Byun should be subjected to a military trial and dismissed disgracefully for self-harming one’s own body.*
	7. She should undergo gender reassignment surgery once again and finish serving as a man	3 (0.5)	*· Even if it’s useless, reconstruct the male genitals with all available medical technique, and let Byun serve in the male troop.*
II. Human rights (*N* = 134)	1. It is impossible to let Byun serve in a female troop because human rights of her female colleagues should be concerned (taking a shower, getting dressed, or sleeping)	76 (13.8)	*· I can fully understand, but the question is whether female soldiers would be willing to share a bedroom and bathroom with Byun.* *· If Byun is allowed in a female troop, THE genuine female soldiers have to get dressed in front of Byun. Don’t they have human rights? This can’t be allowed for god’s sake.*
	2. It is impossible to let Byun serve as a soldier because human rights of her colleagues are violated.	58 (10.6)	*· To respect the human rights of LGBT people might be the demand of the time. Still, the human rights of those who have to work with Byun even if they dislike LGBT people are important.* *· Don’t you know the basic principle of a democratic society? Do no harm to others.*
III. Ineligibility (*N* = 44)	1. She is not mentally eligible since people with gender dysphoria are subject to military exemption (require medical treatment)	25 (4.6)	*· Byun shouldn’t have been allowed in the military in the first place. Letting Byun serve in a female troop doesn’t make a sense at all.* *· It’s a mental disorder! A person suffering from gender identity disorder should not be enlisted in the military. Un expected incidents such as genocide may be possible.*
	2. She is not physically eligible due to loss of male genitals (self-harm)	14 (2.6)	*· There is a military regulation associated with body injury. Any male soldier who has completely lost male genitals are subject to discharge due to the Level three mental and physical disability. If the loss happens during an operation, you receive pension, but in this case, Byun should be discharged due to self-harm.* *· Those with damaged testicles are exempted from the military service.*
	3. She is not physically eligible due to her unchanged genetic characteristics	5 (0.9)	*· Gender reassignment surgery doesn’t change sex chromosome. It is reasonable for the military to review Byun as a disabled person since Byun is a man with an XY paring who has female genitals.*
IV. National perspective (*N* = 33)	1. Military is a specialized organization responsible for maintaining national security of this country which is in a unique situation (truce)	18 (3.3)	*· We’re in a truce right now. Don’t you know what truce is? There is no human rights or consciousness during the war. We’re in a survival game.* *· There is a difference between countries that have national defense obligations and countries that do not.*
	2. She should join the military of another country	10 (1.8)	*· Why can’t it be allowed in this country? You can simply go to another country where it is allowed.*
	3. Female soldiers are useless or weaker than males	5 (0.9)	*· A female soldier or a feminine soldier who should be protected by males wouldn’t be much of help for the national defense.*
VI. Organizational matter (*N* = 17)	1. Military is a specialized organization requiring teamwork.	17 (3.1)	*· Even if we treat Byun as an ordinary person with no prejudice or hatred, there is no doubt that the military is a specialized organization, and a lot of changes should be followed to let Byun serve. We should not only discuss about discrimination against minorities but also about reverse discrimination.* *· There is a clear prejudice against sexual minorities, but this is out of context. This happened in a specialized organization, the military, so that forced discharge is acceptable.*
VII. Undefined (*N* = 172)	1. Presented no specific rationale	101 (18.4)	*· Do I need any explanations? Byun should be dismissed.* *· I am against transgender people joining the military.*
	2. Presented multiple rationale	71 (12.9)	*· A person with unclear gender identity cannot be allowed in the same place with female soldiers. Byun is not qualified as a female soldier since the military enlisted Byun as a male in the first place.* *· The military protects this country, and it is where energetic young people are crowded. A transgender person joins the army and causes a chaos? If your child were to serve with a transgender person, who would support it as a parent? Do homosexuals have human rights while straights have dog rights?*
**Total**		**549 (100.0)**	

^a^Byun had gained an official permission by the supervisor before undergoing gender reassignment surgery

## Discussion

Baker has stated that “transgender people are those whose gender identity differs from that typically associated with the sex they were assigned at birth [11].” Not all transgender people have significant distress, Diagnostic and Statistical Manual of Mental Disorder 5th Edition (DSM-V) uses “gender dysphoria” to clinically describe such distress [11]. During her lifetime, Hee-Soo Byun had stated that she underwent gender affirming surgery just to live as a woman and a soldier [12]. However, South Korea’s current military personnel regulations are not aligned with the definition of transgender presented in DSM-V. It is outdated to view transgender identity as a mental illness [13]. Another hurdle with the inclusion of transgender people in the military service in South Korea is the dichotomous categorization of the human sexes based on anatomy. Discrimination is fundamentally based on the categorization by sex, not gender, so it is difficult for trans people to belong within the organization. If the categorization were based on gender, then Ms. Byun would have belonged to the female soldiers. To include transgender people in the military, the Ministry of National Defense should consider proper preparations regarding physical environments for cisgender and transgender soldiers to co-exists within an organization. As described above, in this study, opposing comments that presented worrisome opinions that the human rights of cisgender people would be in danger if the inclusion of transgender people in the military service is allowed were easily found. Furthermore, a previous study on 70 trans women who were serving in the military and who had completed their obligations at the time of the survey revealed that half of them reported having discomforts in using shower and sleeping facilities with cisgender people [5]. These results indicate that both the general population as well as transgender people pointed out that proper preparations regarding physical environments are necessary for cisgender and transgender to co-exist.

In the present study, we could easily find biased and discriminatory expressions against transgender people in newspaper articles. As part of the analysis, we investigated news article headline keywords to determine whether they discussed Byun’s experience of gender dysphoria. This study found that no article, categorized as “anonymous” had used the term “gender dysphoria” in its headline. However, the term “gender transitioning” was used regardless of the newspapers’ political orientations. A more provocative term, “sex reassignment surgery” was also identified. More than one headline, which addressed Byun’s original sex at birth (male), was confirmed from each newspaper, except for one liberal newspaper. Emcke has emphasized that hate is not individual or sudden, but socially trained and taught [14]. Even though Byun’s supervisor acknowledged and permitted the gender affirming surgery officially, some headlines emphasized the terms “on vacation” and “on duty.” It is presumed that these kinds of terms may have caused misunderstanding by implying that the surgery was performed impulsively or that transgender soldiers are not faithful to their obligations. Furthermore, such biased and discriminatory expressions were used to criticize Byun in comments against transgender soldiers. Additionally, it was found that Byun was often associated with her rank or position in the military even after the forceful discharge. Surprisingly, the proportion of articles that used the term ‘former’ was less than 50%. Some readers’ comments expressed discomfort about this kind of description by saying, “*Why is he still called sergeant*?” Since news reports pursue accuracy and informativeness, it is easily believed that their contents are based on facts [15]. It is not easy for cisgender people to notice their encounters with transgender people in their everyday lives, but it is easier for them to develop a misunderstanding or prejudice about transgender people based on the media’s portrayal of them [15]. Therefore, any form of media, including daily news reports, needs to be responsible and ensure appropriateness and neutrality of words when describing transgender people. Biased and discriminatory expressions against transgender people in mass media should be properly managed according to the Standards for Human Rights reports proposed by the Journalists Association of Korea. This not only infringes upon the dignity and human rights of transgender individuals but also induces hostility toward LGBT individuals, thereby associated problems could be raised following discrimination and prejudice [16].

In this study, we mainly assessed comments on media stories from members of the general public in order to examine attitudes towards transgender soldiers. As expected, the proportion of comments opposing Byun serving in the military in any form was high. Transgender people became publicly known in Korea in 2001 when Risu Ha appeared in the media as an entertainer and gained popularity [17]. Regardless of 20 years’ time difference, the level of social acceptability between Hee-Soo Byun who wanted to serve in the military and Risu Ha, a celebrity, differed. A previous study that analyzed the opinions of 500 people using the 2017 Global Attitudes toward Transgender People Survey revealed that 45.2% of the participants agreed that transgender people should be allowed to serve in the military [18]. This discrepancy might be caused by the differences in characteristics of the respondents/commenters and methodologies. However, caution should be exercised since bold opinions were expressed through online comments due to complete anonymity and not in face-to-face interviews or surveys.

One of the noticeable issues among the comments supporting the inclusion of transgender people in military service was addressing the necessity of re-examining Byun’s current eligibility as a female soldier following the gender affirming surgery. This can be connected to the legitimate employment process indicating that justice and fairness in an employment process are important among Koreans.

Moreover, some of the quotes representing opposing comments reflect not only transphobia, but also sexism and even misogyny. Such comments viewed female soldiers as not helpful for national defense as compared to male soldiers based on their beliefs of physical inferiority of females. However, it is commonly believed that transgender identity does not indicate physical unfitness [19]. In addition, the general population’s lack of understanding about gender-related terms, such as gender identity and gender expression, should be improved. For example, it was found from an opposing comment that sexual orientation was confused with gender identity — “*It should be never allowed. Homosexualism is a type of mental disorder. They should go to the hospital rather than the military*.”

### Study limitations

The present study has several limitations. First, the analysis was only completed for four major daily newspapers. Second, it is likely that the opinions of certain age groups, who were familiar with the use of internet or smartphones, were mainly collected, and therefore this study’s results may not be generalized for other age groups. Third, specific analyses based on the readers’ detailed information were impossible. Nevertheless, it is reasonable to infer that the comments collected in this study reflect the opinions of male readers. Since serving in the military is mandatory for every able-bodied male in Korea, military-related issues mainly attract the attention of males. Fourth, although various opinions were noted, researchers were not able to check whether each of these opinions were scientifically relevant or could be backed with any scientific evidence. Nevertheless, the study results may be valuable since it examined opinions on transgender soldiers in Korea for the first time.

## Conclusions

This study is only a fragmentary result revealing how their existence is filtered by the media’s gaze and how hatred is abetted within the window of freedom. It is expected that these results can serve as a basis for further studies which examine the acceptability of transgender people as members of the society. Moreover, it would also be helpful in the formulation of policies that protect the human rights of the LGBT people.

## Data Availability

The data that support the findings of this study are available from websites of the following newspapers; *Chosun Ilbo* (https://www.chosun.com), *Joongang Ilbo* (https://www.joongang.co.kr), *Hankyoreh* (https://www.hani.co.kr), and *Kyunghyang Shinmun* (https://www.khan.co.kr). The dataset used and analyzed during the current study are available from the corresponding author upon reasonable request.
